# The Discharge Behavior and Mechanism of Polyimide Aerogel under Electron Irradiation

**DOI:** 10.3390/polym16060773

**Published:** 2024-03-11

**Authors:** Dandan Ju, You Wu, Hao Wang, Chengyue Sun, Yiyong Wu, Zhengli Cao, Xueqiang Wang, Guiru Jing, Changjiang Li

**Affiliations:** 1 Laboratory for Space Environment and Physical Sciences, Harbin Institute of Technology, Harbin 150001, China; 2 School of Material Science and Engineering, Harbin Institute of Technology, Harbin 150001, China; 3 Xi’an Aerospace Chemical Propulsion Co., Ltd., Xi’an 710511, China; 4 Shanghai Institute of Aerospace System Engineering, Shanghai 201108, China

**Keywords:** polyimide aerogel, electron irradiation, inhomogeneous charge deposition, anisotropy discharge

## Abstract

The response and mechanism of polyimide aerogel under electron irradiations were investigated. The experimental results indicated that electron irradiation could not damage the skeleton polyimide in the aerogel due to its high stability, but could result in a discharge within. The morphology of the discharge shows some dendritic discharge patterns, and the material surrounding the discharge channels was carbonized. The numerical simulation results indicated that the incident electrons, and also large amount induced secondary electrons, would be deposited inhomogeneously within the nano-porous polyimide aerogel. This would result in forming an ultra-high electrical potential of up to about 8.5 × 10^10^ V/m (which is far higher than the breakdown strength (2 × 10^8^ V/m) of bulk polyimide materials) in a local region. This may be the leading cause of the obvious discharge in the materials. Furthermore, it was found that the actual reason for the discharge is related to the residual gas within the nano-porous structure; namely, the more internal residual gas (as a shorter-time vacuum pumping in the irradiated chamber), the more serious the discharge phenomenon. Correspondingly, the phenomenon may largely consist of both residual-gas discharge and surface flashover due to ultra-high local potentials induced by unevenly deposited charges in the porous aerogel.

## 1. Introduction

With the exploration and development of the aerospace industry, the materials used under extreme environments have received extensive attention [1]; they require the properties of being ultra-light, flexible, deployable [2], irradiation-resistant, and temperature-resistant. Aerogels [3] are a novel material, with three-dimensional open networks assembled by coherent nanoparticles, large specific surface areas, and high porosities, typically greater than 90%. The extremely low density and excellent thermal/sound insulation performance [4] meant that aerogels exhibit great potential for aerospace activities [5].

Silica aerogel has been used as a space hyper-velocity dust capturer [6] due to its extremely high porosity, transparency, and nano-skeleton structure. In the 2003 Mars Exploration Rovers (Spirit and Opportunity), this aerogel was used as a thermal insulation material in the Warm Electronics Boxes (WEB) [6]. However, the brittleness of inorganic aerogel limits its extensive application in the aerospace field [7]. Comparatively, the flexible polyimide (PI) aerogel is an attractive prospect for extravehicular activity (EVA) suits and thermal protection systems (TPSs) for inflatable aerodynamic decelerators, and its thermogravimetric temperature can reach 300~400 °C [8,9]. In addition, the PI aerogel also exhibits a broader bandwidth and higher gain when used as an antenna substrate [10]. Materials used in space are exposed to various types of radiation from the space environment, such as protons, electrons, plasma, cosmic rays and ultraviolet rays, etc. The proton and electron irradiation would cause displacement and ionization effects in polymers and result in scission or crosslinking between molecular chains [11,12]. In addition, the surface and internal discharge phenomenon [13] in the insulation materials (such as polymers) under electron irradiation also has a catastrophic effect on spacecraft. Although there have been plenty of reports on the damage behavior and mechanisms of bulk/film polyimides under various particles’ irradiation [13,14,15,16,17], the influence of a three-dimensional porous structure and nanoscale skeleton on radiation effects is still unclear. There have been several studies on the effect of proton irradiation [18,19,20] on polyimide aerogels. The Monte Carlo simulation results indicated that a porous structure could enhance the proton-scattering effects due to a higher specific surface area and more boundaries [18]. After proton irradiation, the three-dimensional network of an aerogel would become densified and even transform into a layer-by-layer structure [19]. The optical absorption increased and the specific heat capacity decreased linearly with proton fluences, which can be attributed to irradiation damage and carbonization in polyimide aerogels [20].

While there is little research on the damage effect of organic aerogels under electron irradiation, in this work, the transport behaviors of electrons in nano-porous polyimides were simulated to make clear whether the nano-porous structure takes on special roles in the effects of irradiation on aerogels. In addition, the responses and mechanisms of polyimide aerogels under electron irradiation were investigated by testing. The results will contribute to revealing the radiation effects and damage mechanisms of organic porous materials.

## 2. Materials and Methods

### 2.1. Materials

**Figure 1:**
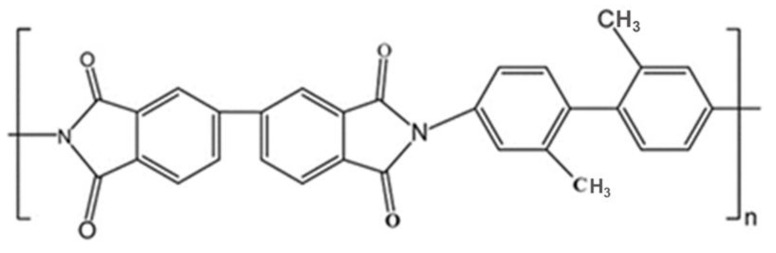
The chemical structure of the repeating unit of the PI aerogel.

In this study, the polyimide (PI) aerogel was obtained from Aerospace System Engineering (Shanghai, China). It was synthesized through the co-polycondensation of 3,3,4,4′-biphenyltetracarboxylic dianhydride (s-BPDA) and 2,2-dimethylbenzidine (DMBZ), followed by the application of the supercritical carbon dioxide drying treatment. The synthesis details are described elsewhere [21] and the chemical structure is shown in Figure 1. The apparent density of the PI aerogel is 1.4 g/cm^3^. Eventually, the PI aerogel was cut into rectangular blocks (20 mm × 20 mm × 5 mm and 10 mm × 10 mm × 2 mm) for electron irradiation. The larger block samples (20 mm × 20 mm × 5 mm) were used to explore the influence of internal residual gas on the discharge behavior, while the smaller samples (10 mm × 10 mm × 2 mm) were used to investigate the effects of fluence.

**Figure 2:**
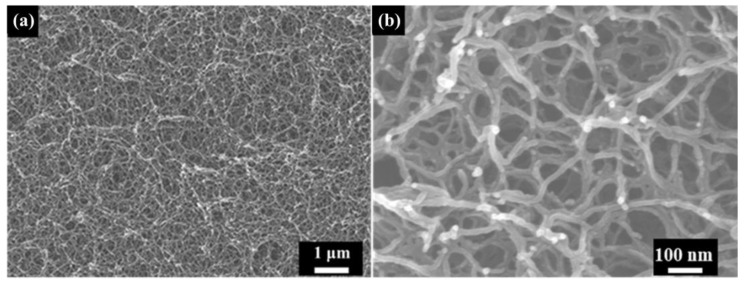
The SEM images of as-received polyimide aerogel: (**a**) Low magnification; (**b**) High magnification.

Figure 2 shows the structural characteristics of the as-received polyimide aerogel. The morphology of polyimide aerogel is a three-dimensional network structure, consisting of nano-pores and nano-scale skeletons (Figure 2a,b). The diameter of the skeletons is about 20 nm, and they are interconnected by nodes.

**Figure 3:**
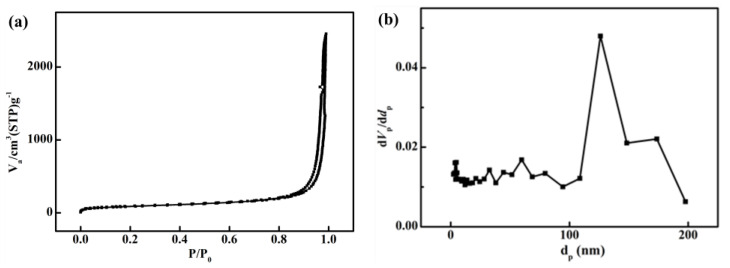
Characterization of pores of polyimide aerogels: (**a**) Nitrogen adsorption isotherms; (**b**) Pore size distributions.

Figure 3a,b show the N_2_ adsorption/desorption isotherms and the calculated pore-size distributions of the as-received polyimide aerogel, respectively. It can be observed that the original polyimide aerogel exhibits a type IV adsorption curve, indicating mesoporous characteristics, with a specific surface area (BET) of 380 m^2^/g. The pore size distribution indicates that most pores fall within the size range of 0 to 200 nm, with an average pore size, calculated by the BJH model, of 51 nm.

### 2.2. Electron Irradiation

The electron irradiation tests of PI aerogel were performed at room temperature using the КИФК integrated irradiation simulator, located in the National key Laboratory of Space Environment Material Behavior and Evaluation Technology of the Harbin Institute of Technology. The specific irradiation parameters are shown in Table 1.

**Figure 4:**
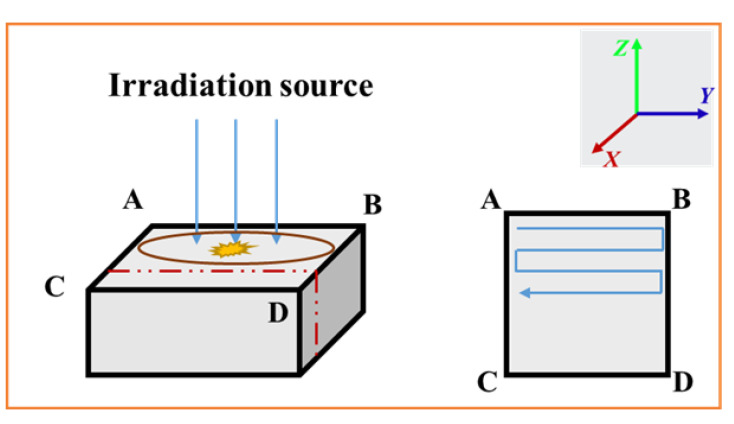
Schematic diagram of electron irradiation scanning method: ABCD is the irradiated plane, which is perpendicular to the irradiation direction and the red dotted line is the cross-section parallel to the irradiation direction.

The samples were placed on the holder with the planes of 20 mm × 20 mm and 10 mm × 10 mm perpendicular to the electron beams. The scanning method of the electron beam is Z-shaped, as shown in Figure 4.

### 2.3. Characterizations

After electron irradiation, the surface morphologies were observed using an OLYMPUS microscope. The cross-sectional morphologies were inspected using the Merlin Compact scanning electron microscope (SEM). The cross-section observation samples were prepared according to the red dotted line as shown in Figure 4. The samples were coated with a platinum film for conductivity before SEM observation.

The nitrogen adsorption-desorption isotherm curves were obtained using a 3H-2000PS instrument. The measured samples were outgassed at 80 °C for 8 h under vacuum before nitrogen isotherm adsorption tests. The specific surface area was estimated using the Brunauer–Emmett–Teller (BET) model. The pore size distribution for mesopores was calculated from the desorption branch of the isotherm using the Barrett–Joyner–Halenda (BJH) model. The pore size distribution for macrospores was analyzed using a Micrometrics Autopore IV 9500 mercury porosimeter. The skeleton density of the material was obtained using the helium specific gravity method, with the test equipment being a 3H-2000TD1 automatic true density meter. The test gas was helium, and each sample was tested 6 times.

The internal morphologies (microvoids) of the irradiated samples were studied using a three-dimensional X-ray microscope (3D-XRM, Xradia 520 Versa, German Carl Zeiss) with a spatial resolution of 700 nm. The shape of the 3D-XRM sample was rectangular blocks with dimensions of 2 mm × 2 mm × 2 mm. The main scanning parameters of the XRM test included a voxel size of 0.5263 μm and an object of 2028. The XRM analysis focused on pore shapes and orientation characteristics.

### 2.4. Simulation Software

Geant4 is a Monte Carlo application toolkit developed by CERN (European Organization for Nuclear Research). It is used in this work to simulate the physical process of electron transport behavior in the nanoporous polyimide.

## 3. Results and Discussion

### 3.1. The Influence of Irradiation Fluence on Discharge Behavior

**Figure 5:**
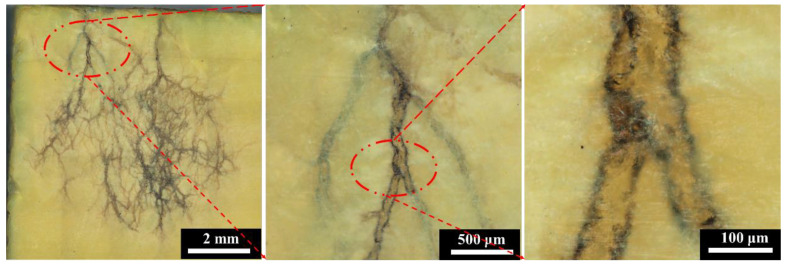
The optical micrograph of polyimide aerogel after 90 keV electron irradiation (the irradiation fluence is 2 × 10^15^ cm^−2^, and the flux is controlled at 1 × 10^12^ cm^−2^s^−1^).

In order to explore the internal discharge behavior and mechanism of the polyimide aerogel under electron irradiation, the morphology of the aerogel was observed and analyzed at different irradiation fluences. Figure 5 illustrates the optical micrograph of the surface of the PI aerogel after 90 keV 2 × 10^15^ cm^−2^ electrons irradiation. The surface is perpendicular to the incident direction of the electrons, and the scanning method of electron is Z-shaped from top to bottom (Figure 4). It can be observed that a dendritic discharge pattern appears in the PI aerogel after electron irradiation, with the dendritic orientation from top to bottom. The color of dendritic discharge tracks has transformed into black, while the material wrapped around the channel remained yellow. The internal discharge morphology in the PI aerogel is similar to that of bulk polymer materials after dielectric breakdown [22].

**Figure 6:**
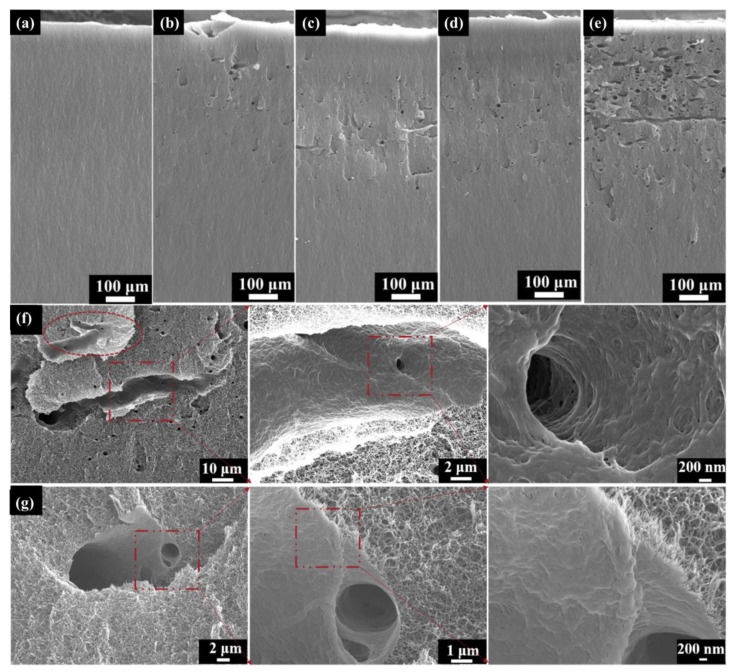
SEM cross-section images of PI aerogel: (**a**) as-received; (**b**) 2 × 10^14^ cm^−2^; (**c**) 5 × 10^14^ cm^−2^; (**d**) 1 × 10^15^ cm^−2^; (**e**) 2 × 10^15^ cm^−2^; (**f**) Typical morphology of discharge channel at the fluence of 2 × 10^14^ cm^−2^ (Parallel to the cross-section); (**g**) Typical morphology of discharge channel at the fluence of 2 × 10^14^ cm^−2^ (perpendicular to the cross-section).

Figure 6 depicts the SEM cross-section images of the PI aerogels under different fluence electron irradiation. For all irradiated samples, the morphological characteristics are completely different from the as-received samples (Figure 6a). Several micrometer-size voids appear at the fluence of 2 × 10^14^ cm^−2^ (Figure 6b). Moreover, the number of voids increases with fluence (Figure 6b–e). The orientation of the voids is mostly perpendicular to the cross-section, while fewer discharge channels are parallel to the cross-section (Figure 6e). To better understand this phenomenon, the typical morphology of the discharge channel at the fluence of 2 × 10^14^ cm^−2^ was observed, as shown in Figure 6f (parallel to the cross-section) and Figure 6g (perpendicular to the cross-section). The tree-like discharge channel (inside the dashed oval and the dashed rectangle) occurred in the cross-section (Figure 6f,g). The three-dimensional network structure of the original PI aerogel has been completely transformed into a densely welded structure in the discharge channel. Previous research has shown that the temperature in the channel caused by partial discharge can reach up to 1000 °C [23], and the polyimide would melt, even vaporize, and transform into an amorphous carbonized structure. It is worth noting that the material wrapped around the discharge channel retains the three-dimensional network structure.

**Figure 7:**
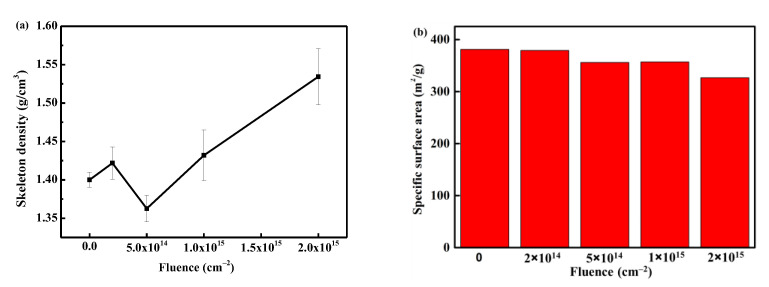
The evolution of skeleton density (**a**) and specific surface area (**b**) with fluence.

In order to explore the specific changes in the skeleton structure, the results of skeleton density and specific surface area with fluence are shown in Figure 7. It can be observed that the skeleton density increases with the increase in fluence. This can be attributed to two factors: (i) the PI aerogel is obtained by supercritical CO_2_ drying, and its skeleton contains a certain amount of CO_2_ molecules. Electron irradiation causes a local temperature rise, leading to the desorption of gas molecules from the skeleton, and the materials around the discharge path are squeezed and densified; (ii) the material on the discharge track carbonizes after reaching high temperatures (above 1000 °C [23]). The skeleton density of the material after carbonization is greater than that of the original PI molecular chain, resulting in an overall increase in material density. These two factors together contribute to the increase in skeleton density.

Figure 7b shows the evolution of the specific surface area of aerogel with the fluence after electron irradiation. The specific surface area exhibits a decreasing trend with increasing fluence. After irradiation with 2 × 10^15^ cm^−2^, the specific surface area decreases from the original 380 m^2^/g to 330 m^2^/g, a decrease of 13%. The decrease in the specific surface area of the aerogel is related to the discharge phenomenon. The original aerogel is a three-dimensional nano skeleton material, and the material at the discharge position is squeezed into a cylindrical pore wall (Figure 6f), with most of the nano-skeleton on the pore wall welded together (Figure 6g). Such structural transformation leads to a decrease in the specific surface area of the material.

### 3.2. The Influence of Irradiation Flux on Discharge Behavior

**Figure 8:**
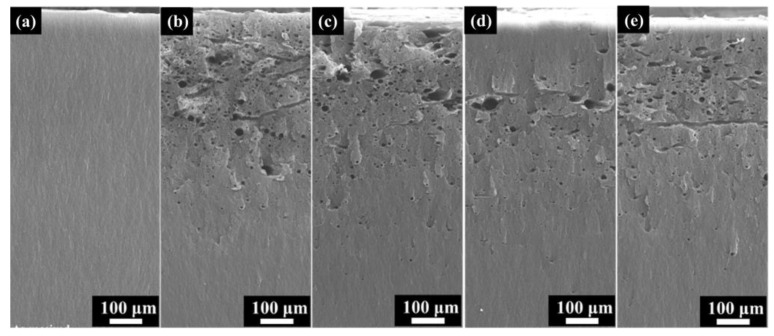
The cross-section SEM morphology after different electron flux irradiation. (**a**) As-received; (**b**) 1 × 1011 cm^−2^s^−1^; (**c**) 3 × 1011 cm^−2^s^−1^; (**d**) 6 × 10^11^ cm^−2^s^−1^ (**e**) 1 × 10^12^ cm^−2^s^−1^.

Figure 8 depicts the SEM morphology of the cross-section after electron irradiation with different fluxes. After electron irradiation, obvious micron-scale holes appear on the cross-section, with most holes oriented perpendicular to this section. There are also channels parallel to the cross-sections with fluxes of 1 × 10^11^ cm^−2^s^−1^ and 1 × 10^12^ cm^−2^s^−1^. This indicates that the holes generated by discharge have two directions. However, the two-dimensional morphology may not fully reflect the internal pore structure of the material. Therefore, a three-dimensional morphology analysis of discharge channels will be conducted in Section 3.4.

**Figure 9:**
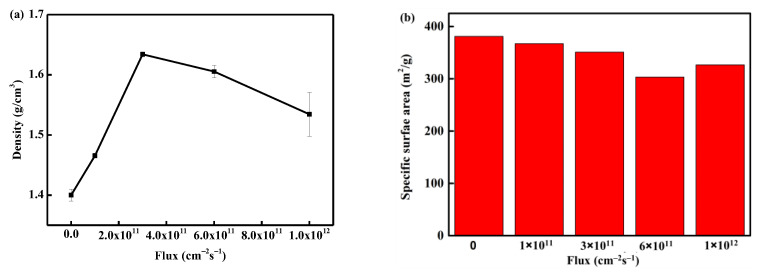
The evolution of skeleton density (**a**) and specific surface area (**b**) with flux.

Figure 9 illustrates the evolution of the skeleton density and specific surface area with flux after electron irradiation. The skeleton density initially increased, then slightly decreased, and eventually showed a saturation trend with the increase of flux. However, the specific surface area gradually decreased with the increase of flux and exhibited a trend of saturation. The evolution of the skeleton structure indicates that an increase in flux will elevate the discharge risk. With a larger electron beam, more discharge paths are formed. A detailed analysis of the specific mechanism will be provided in Section 3.5.2.

### 3.3. The Influence of Residual Gas on Discharge Behavior

**Figure 10:**
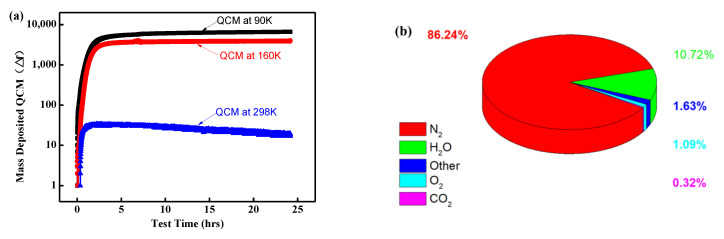
Outgassing evolution characteristic of polyimide aerogel: (**a**) The vibration frequency of the microbalance with time at different temperatures; (**b**) The gas composition ratio.

Due to the high specific surface area of aerogel, which means a higher free surface area, more gas molecules can be adsorbed in the ambient environment. In order to evaluate the effect of surface gas adsorption on the discharge behavior, the outgassing behavior of the aerogel in a vacuum was analyzed and mass spectrometry performed. In this study, the ASTM E1559 [24] standard was utilized to investigate the outgassing behavior of PI aerogel under vacuum conditions. Quartz microbalances at 90 K, 160 K, and 298 K were placed above the samples. The evolution of condensing volatiles over time was recorded by the quartz microbalance. Figure 10a depicts the relationship between the frequency vibration of the microbalance and the outgassing time of the PI aerogel at different temperatures. It can be observed that the vibration frequency follows a similar pattern at different temperatures, initially increasing over time, and then tending to saturate after 2.5 h. However, the vibration frequency varies significantly at different temperatures (90 K, 160 K, and 298 K), indicating that the gas volatiles of PI aerogel are temperature-sensitive. Consequently, a mass spectrometer was used to analyze the outgassing components of the PI aerogel, as depicted in Figure 10b. It can be observed that the outgassing components mainly consist of nitrogen (86.24%), oxygen (1.09%), water molecules (10.72%), and carbon dioxide (0.32%). These results indicate that the composition of the gas adsorbed in polyimide aerogel is similar to that of air.

**Figure 11:**
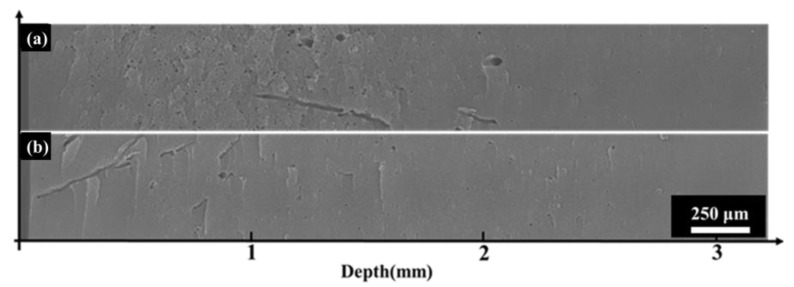
The influence of pumping time on irradiation effect (the left side is the direction of electron incidence). (**a**) 0.5 h; (**b**) 9 h.

Figure 11 illustrates the cross-sectional morphology of polyimide aerogel after 170 keV electron irradiation under different pumping times (the fluence is set at 2 × 10^15^ cm^−2^. The pumping times are 0.5 h and 9 h, respectively). It is evident that the morphology exhibits distinct differences at different pumping times. When the pumping time was 0.5 h, dense micron-level circular holes and a few dendritic holes appeared in the cross-section. When the pumping time was 9 h, dendritic and circular holes also appeared on the cross-section, but their number and area were significantly fewer and smaller compared to the shorter pumping time (0.5 h). These results indicate that polyimide aerogel would exhibit an obvious discharge phenomenon under electron irradiation, and the degree of discharge is related to the internal residual gas. The more internal residual gas present, the more severe the discharge phenomenon. Moreover, there are two main discharge orientations: one perpendicular to the cross-section (the circular holes), and the other approximately parallel to the cross-section (dendritic holes). The discharge behavior and mechanisms will be discussed in Section 3.5.

### 3.4. Analysis of Discharge Channel Anisotropy

**Figure 12:**
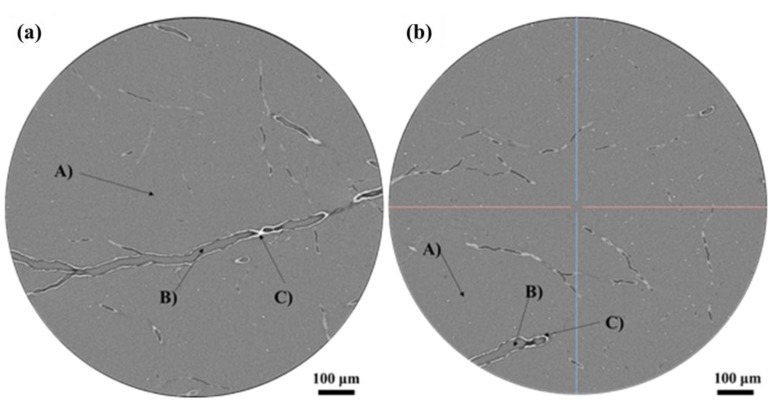
Three-dimensional-CDT (3D-CT) slice morphology (these slices are parallel to the X-Y plane in Figure 4). (**a**) Thicker dendritic discharge channel; (**b**) Thinner dendritic discharge channel.

As discussed above, there are two main discharge orientations (Figure 6 and Figure 8): one perpendicular to the cross-section (the circular holes) and the other approximately parallel to the cross section (scratch-like and dendritic holes). In order to better analyze the discharge orientation, the coordinate axes in Figure 4 are taken as the reference system, and the 3D-CT is used to reconstruct the discharge channel. Figure 12 illustrates the morphology of the slice with an irradiation fluence of 2 × 10^14^ cm^−2^. These slices are parallel to the X-Y plane in Figure 4. Figure 12a depicts the morphology of the thicker branches in these slices, while Figure 12b shows thinner branches. It can be observed that the slice morphology can be divided into three distinct phase regions: A, B, and C. Part A is a mixed phase, consisting of polyimide aerogel nano-ligaments and air (the space resolution of 3D-CT is about 0.56 microns, and these two phases cannot be distinguished). Part B represents the air phase, while part C represents the welded nano-ligament of polyimide aerogel. Moreover, the proportion of phase C is quite small, indicating that the discharge occurs only in a localized area of the polyimide aerogel.

**Figure 13:**
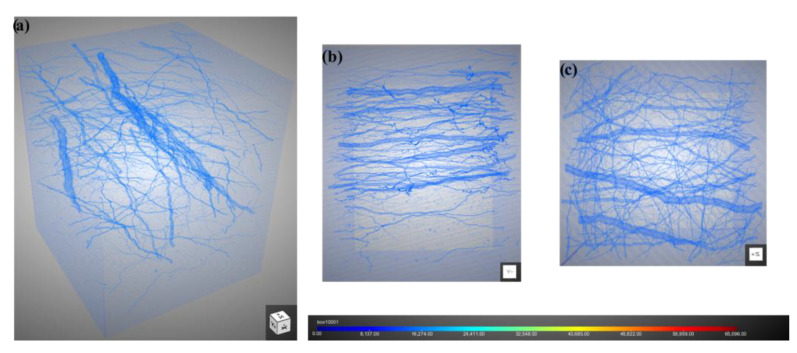
Reconstruction of discharge channel: (**a**) three-dimensional perspective; (**b**) lateral view (Y direction); (**c**) top view (Z direction).

In order to observe the three-dimensional internal discharge morphology more clearly, slices at the fluence of 2 × 10^14^ cm^−2^ were reconstructed, as shown in Figure 13. It can be observed that the dendritic discharge channels (Figure 13a) are mostly perpendicular to the Z direction (the direction of electron incidence). The orientation of thicker branches is nearly parallel to the X direction. Figure 13b,c show the lateral and top views of the discharge channel in the Y and Z directions, respectively. It is worth noting that that orientation of the thicker branches is predominantly parallel to the X direction (Figure 13b), while the orientation of thinner branches is relatively random as a whole (Figure 13c). These results indicate that there is a larger potential difference in the direction of the X compared to the Y axis during the electron irradiation process.

**Figure 14:**
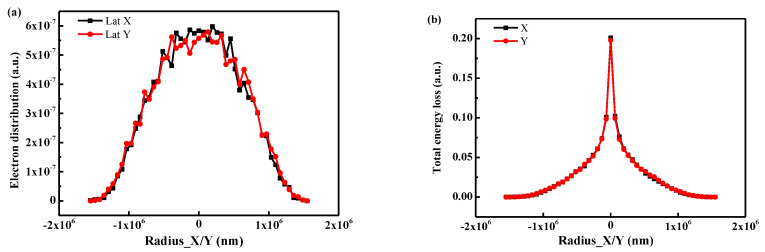
The lateral electron transport statistics calculated by the software Win-X-Ray (1.4.2.1) around the incident point in the lateral direction of X/Y: (**a**) the electron distribution; (**b**) the energy loss.

In order to determine the influence of the electron transport process on the anisotropy discharge, the transport results of the incident electron in the direction of X/Y were calculated, as shown in Figure 14. It can be observed that there is no obvious difference in the distribution curves of electron and energy loss in the X/Y directions. The results indicate that the electron transport process shows little effect on anisotropy discharge. The anisotropy discharge channel is related to the electron irradiation control scanning mode. The scanning mode of the electron beam is Z-shaped (Figure 4), resulting in a time difference in charge accumulation. More specifically, the time lag in the X direction is much larger than in the Y direction, leading to the distinct anisotropic discharge channel.

### 3.5. Discussion of Discharge Mechanism

#### 3.5.1. The Simulation of Electron Transport Behavior into Nanoporous Polyimide

**Figure 15:**
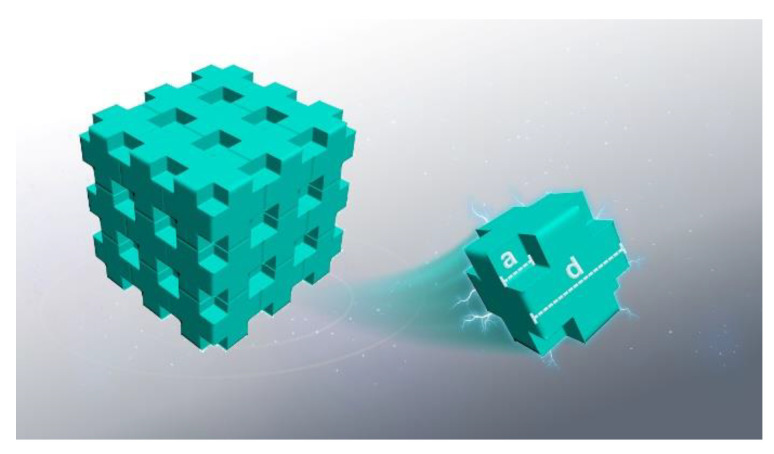
The schematic diagram of cubic periodic nanoporous structure.

Based on the structure characteristics of the as-received PI aerogel, a simple model was proposed to simulate the electron transport behavior in nanoporous PI, as shown in Figure 15. Considering that the porous materials consist of cubic-shaped pores homogeneously and periodically distributed in a solid, the distance between adjacent voids is defined as d, and the length of voids’ side is set as a. The different porosities corresponding to the values of a and d are shown in Table 2. In this study, the electrons energy is set at 5 keV, and the number of incident electrons is set at 200,000 for different porosities. In this scenario, the incident electrons should transport straightly within the voids. Using the above-defined scaling parameters, the porosity of the defined materials can be calculated using the formula (1):

(1) $$\eta =\frac{8\cdot a^{3}}{d^{3}}.$$

**Figure 16:**
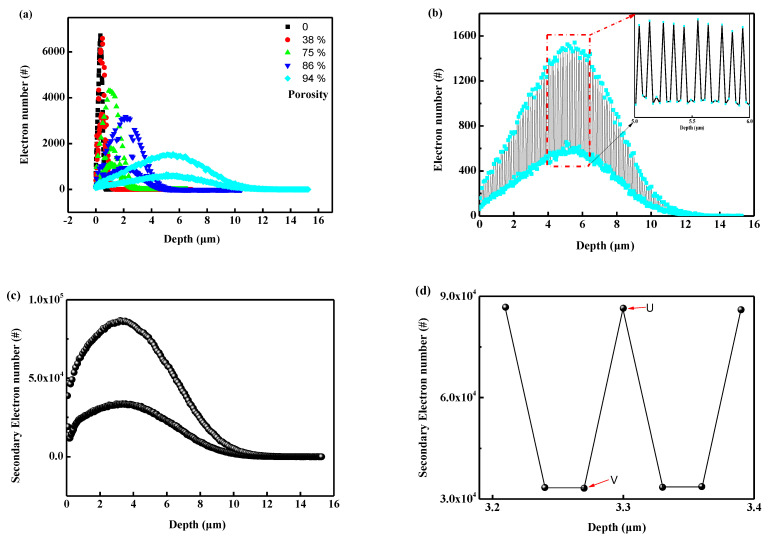
The transport results calculated by Geant4: (**a**) the distribution of incident electron at different porosity; (**b**) the incident electron distribution at the porosity of 94%; (**c**) the secondary electron distribution at the porosity of 94%; (**d**) the local zoom view of (**c**).

Figure 16 illustrates the transport behavior of 5 keV electrons in nanoporous PI. Figure 16a depicts the incident electron distribution at different porosities. Several features can be observed with increasing porosity. Firstly, the average range and FWHM (full width at half maxima) of the incident electrons increase with porosity. The average range is 0.32 μm for bulk PI and 5.6 μm at 94% porosity, respectively. Moreover, distinct envelopes appeared in the incident electron distribution curves, becoming more pronounced with increasing porosity. To better understand this phenomenon, the distribution curve of incident electrons at 94% porosity is isolated, as shown in Figure 16b. It can be observed that the number of incident electrons first increases to a peak, then decreases to a valley, and continues to distribute along the depth. Importantly, the distance between adjacent peaks and valleys exhibits a certain periodicity. The non-uniformity distribution of the incident electrons is related to the nanoporous geometry in Figure 10. Specifically, the peaks appear at the center of two adjacent voids, where the amount of material is greater than that of the voids in the incident direction. Figure 16c depicts the distribution of secondary electrons along the incident direction. The distribution pattern is similar to that of the incident electrons, but the total number of secondary electrons is approximately 60 times greater than that of incident electrons. Compared to the distribution of the incident electrons, the secondary electrons trend to move towards the surface (the center of the secondary electron distribution curve is located at 3.2 μm, while the center of the incident electron is at 5.6 μm). Figure 16d provides a local magnified image of Figure 16c at a depth of 3.3 μm. It can be observed that the distribution of secondary electrons is noticeably uneven in local areas. The electrical potential between points U(3.3, 86,450) and V(3.27, 33,192) is up to 8.5 × 10^10^ V/m, which is significantly higher than the breakdown voltage (2 × 10^8^ V/m) of bulk polyimide materials [25]. These results indicate that the uneven distribution of a large number of secondary electrons in porous materials increases the risk of discharge.

The field strength between points U and V can be calculated using the following formula:

(2) $$E_{12}=\frac{kQ_{1}}{r^{2}}-\frac{kQ_{2}}{r^{2}}=\frac{k}{r^{2}}\left(Q_{1}-Q_{2}\right)=\frac{kq}{r^{2}}\left(n_{1}-n_{2}\right).\;$$

*E*_12_: The field strength between U and V;

*k*: The electrostatic force constant, 9.0 × 10^9^ N·m^2^/C^2^;

*q*: The elementary charge, e = 1.6 × 10^−19^ C;

*r*: The distance of U and V;

*n*_1_: The number of charge at U;

*n*_2_: The number of charge at V.

#### 3.5.2. Mechanisms of Flux Influence on Discharge Behaviors

As discussed in Section 3.2, under the same conditions of incident electron energy and fluence, higher flux leads to more severe internal discharge, lower specific surface area, and higher skeleton density. The influence of flux on these factors can be analyzed from the perspectives of charge deposition and annihilation. Several hypotheses are proposed:

(1) For a given incident electron energy, its transport behavior in the material is determined. With a fixed number of incident electrons (200,000 in this case), the difference in secondary electrons between point U and point V is 53,258 in Figure 16c; this implies that, for every 100 incident electrons, there is a difference of 26 electrons between U and V. The amount of secondary electron deposition at points U and V is proportional to the number of incident electrons.
(2) When secondary electrons are deposited into the nano-skeleton, the annihilation rate remains constant (denoted as k) and does not change with the flux.
(3) The local charge difference between points U and V equals the difference between the secondary electrons and the annihilation part.
(4) The breakdown strength of the material is denoted as E. When the electric field strength at points U and V equals the breakdown strength E, discharge occurs and the accumulated charge on the skeleton is completely released.

**Figure 17:**
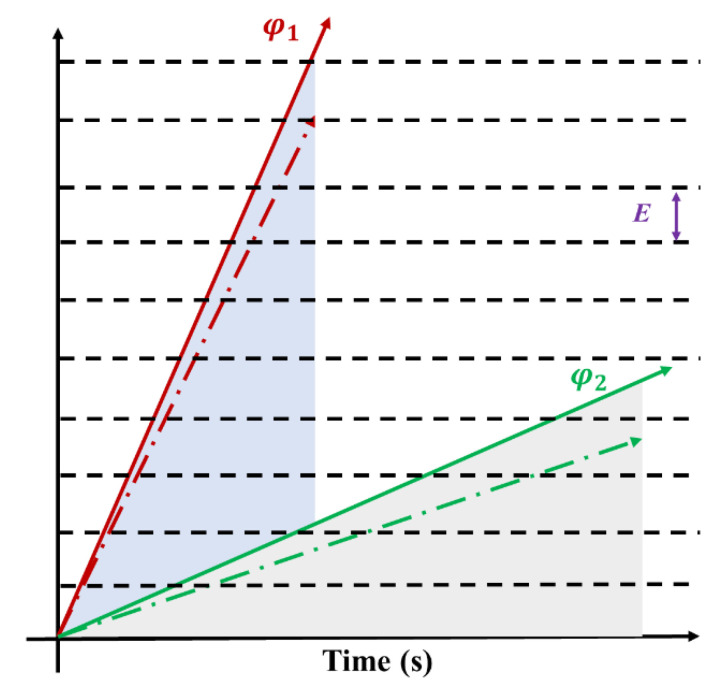
The schematic diagram of discharge effect at different fluxes.

Figure 17 illustrates the charging process under different irradiation fluxes. φ_1_ represents the charge accumulation rate of high-flux electron irradiation (solid line), while φ_2_ represents the charge accumulation rate of low-flux electron irradiation (solid line). Both curves show a linear increase trend over time, and the area enclosed by both curves and the horizontal axis time represents the same fluence. With the increase of irradiation time, charge deposition creates a potential difference within the material, while charge annihilation weakens it. When the discharge threshold is reached, the discharge occurs. The establishment process of the potential difference is depicted by the dashed lines near φ_1_ and φ_2_ in Figure 17 (the slope equals the rate of charge accumulation minus the rate of annihilation). The distance between the black dashed lines in Figure 17 represents the discharge threshold E. When the charge accumulation reaches the black line, discharge occurs. It is worth noting that high-flux electron irradiation intersects with the black dashed line more frequently, indicating more discharge occurrences during the irradiation process. Higher flux leads to a faster charge deposition rate, easier establishment of charge difference, and shorter time to reach the discharge threshold. In summary, higher flux results in more severe discharge damage, consistent with experimental findings.

#### 3.5.3. Discharge Mechanism Analysis

**Figure 18:**
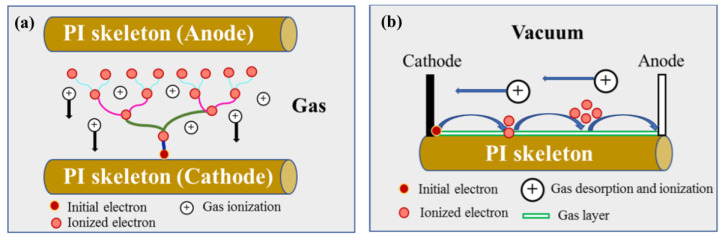
The schematic diagrams of discharge type: (**a**) gas discharge; (**b**) surface flashover.

Based on the simulation results, it can be inferred that the nanoporous structure of aerogel significantly alters the distribution of electrons in the material. Moreover, the distribution of secondary electrons creates an extremely high electric field in local areas. Indeed, the electrical potential between points U and V reaches up to 8.5 × 10^10^ V/m (with only 20,000 incident electrons, and the minimum fluence used in the irradiation test being 2 × 10^14^ cm^−2^, far exceeding the number of simulated incident electrons), which far exceeds the breakdown strength (2 × 10^8^ V/m) of the bulk polyimide materials. The unique structure (nano-skeleton and nano-porous) significantly increases the discharge risk of polyimide aerogel under electron irradiation. What is more, gas adsorption on the nano-skeleton surface reduces the dielectric constant of material, exacerbating the discharge situation. In addition, the influence of residual gas on discharge behavior indicates that the discharge type is correlated with the gas. Specifically, there are two main discharge types: gas discharge and surface flashover, as illustrated in Figure 18. The uneven deposition of secondary electrons on the nano-skeleton creates a local potential difference. In this study, the nano-skeleton with more secondary electron deposition acts as the cathode, while the other side serves as the anode. For gas discharge (Figure 18a), the secondary electrons on the cathode move towards the anode under the influence of the local electric field. During this movement, they collide with and ionize the residual gas, forming an electron avalanche. The newly generated particles (electrons and ions) then move towards both sides of the electrode under the acceleration of the electric field. This process is a positive feedback loop, and the electronic avalanche forms a conduction path (discharge). As the pumping time increases, the residual gas content gradually decreases, and the discharge type transitions to the surface flashover, as shown in Figure 18b. Surface flashover typically occurs at the interface between the gas and the nano-skeleton surface, and the process is similar to that of the gas discharge. Both of these discharge types involve gas, which plays an important role in the discharge process. The presence of gas tends to lower the discharge threshold compared to the dielectric discharge. In this study, with a nano-skeleton diameter of only 20 nm and the farthest distance of the electron to the surface being 10 nm, electrons produced during the irradiation process can easily reach the nano-ligament surface due to the significant surface driving force. The likelihood of discharge in the medium (nano-skeleton) is extremely low. Based on the aforementioned results and discussion, the discharge types in PI aerogel are primarily determined by the presence of gas: gas discharge and surface flashover.

## 4. Conclusions

The transport behaviors of incident electrons in nanoporous matter and discharge effects were investigated. Through this study, the following conclusions can be drawn:

1. The electron irradiation induces internal discharge in the PI aerogel, forming dendritic discharge channels and micrometer-scale pores. The specific surface area decreases with the increase of fluence and flux. Dense tissue forms around the discharge channel due to the welding effect during the discharge process. Higher flux increases the discharge risk by creating more discharge paths. Moreover, the degree of discharge and the discharge type are related to the internal residual gas content. Greater residual gas leads to a more severe discharge phenomenon.
2. The transport behaviors of electron in nanoporous matter were investigated, revealing local inhomogeneity in electron distribution due to the presence of voids. The incident electron number initially increases to a peak, then decreases to a valley, and continues to distribute along the depth. The distance between adjacent peaks and valleys exhibits periodicity. The non-uniformity distribution of the incident electrons is influenced by nanoporous geometry, resulting in an extremely high electrical potential (up to 8.5 × 10^10^ V/m), surpassing the breakdown strength (2 × 10^8^ V/m) of bulk polyimide materials.
3. The anisotropy discharge channels form after electron irradiation, attributed to the scanning mode of electron control. The Z-shaped scanning mode introduces a time difference in the charge accumulation process. Under identical incident electron energy and fluence, higher flux accelerates charge deposition, facilitates charge difference establishment, and shortens the time to reach the discharge threshold. Consequently, higher flux exacerbates discharge damage.

Since discharge can lead to serious failures in spacecraft systems, preventive measures should be implemented to mitigate the discharge phenomenon when using aerogel in spacecraft. Possible measures include enhancing the external protective layer of polyimide aerogel to block electron irradiation injection or controlling the porous structure of the aerogel to reduce discharge occurrence. These strategies will be further investigated in future studies.

## Figures and Tables

**Table 1:** Parameters of polyimide aerogel electron irradiation.

Electron Energy (keV)	Fluence (cm^−2^)	Flux (cm^−2^s^−1^)	Sample Size (mm × mm × mm)	Pumping Time (h)
170	2 × 10^15^	1 × 10^12^	20 × 20 × 5	0.5
				9
90	2 × 10^14^	1 × 10^12^	10 × 10 × 2	3
	5 × 10^14^			
	1 × 10^15^			
	2 × 10^15^			
90	2 × 10^15^	1 × 10^11^	10 × 10 × 2	3
		3 × 10^11^		
		6 × 10^11^		

**Table 2:** The different porosities correspond to the values of a and d.

a (nm)	d (nm)	Porosity
50	102	94%
50	105	86%
50	110	75%
50	138	38%

## Data Availability

Data are contained within the article.

## References

[1] Beyerlein I.J., Caro A., Demkowicz M.J., Mara N.A., Misra A., Uberuaga B.P. (2013). Radiation damage tolerant nanomaterials. Mater. Today.

[2] Hou L., Wu Y., Xiao J., Guo B., Zong Y. (2019). Degeneration and damage mechanism of epoxy-based shape memory polymer under 170 keV vacuum proton irradiation. Polym. Degrad. Stab..

[3] Du A., Zhou B., Zhang Z., Shen J. (2013). A Special Material or a New State of Matter: A Review and Reconsideration of the Aerogel. Materials.

[4] Xu X., Zhang Q., Hao M., Hu Y., Lin Z., Peng L., Wang T., Ren X., Wang C., Zhao Z. (2019). Double-negative-index ceramic aerogel for thermal superinsulation. Science.

[5] Randall J.P., Meador M.A., Jana S.C. (2011). Tailoring mechanical properties of aerogels for aerospace applications. ACS Appl. Mater. Interfaces.

[6] Jones S.M. (2006). Aerogel: Space exploration applications. J. Sol-Gel Sci. Technol..

[7] Guo H., Meador M.A., McCorkle L., Quade D.J., Guo J., Hamilton B., Cakmak M. (2012). Tailoring properties of cross-linked polyimide aerogels for better moisture resistance, flexibility, and strength. ACS Appl. Mater. Interfaces.

[8] Guo H., Dewey O.S., McCorkle L.S., Meador M.A.B., Pasquali M. (2019). Polyimide Aerogels as Lightweight Dielectric Insulators for Carbon Nanotube Cables. ACS Appl. Polym. Mater..

[9] Pantoja M., Boynton N., Cavicchi K.A., Dosa B., Cashman J.L., Meador M.A.B. (2019). Meador, Increased Flexibility in Polyimide Aerogels Using Aliphatic Spacers in the Polymer Backbone. ACS Appl. Mater. Interfaces.

[10] Meador M.A., Wright S., Sandberg A., Nguyen B.N., Van Keuls F.W., Mueller C.H., Rodriguez-Solis R., Miranda F.A. (2012). Low dielectric polyimide aerogels as substrates for lightweight patch antennas. ACS Appl. Mater. Interfaces.

[11] Sun C., Wu Y., Yue L., Shi Y., Xiao J. (2012). Investigation on the recombination kinetics of the pyrolytic free-radicals in the irradiated polyimide. Nucl. Instrum. Methods Phys. Res. Sect. B.

[12] Sun C., Wu Y., Xiao J., Li R., Yang D., He S. (2011). Pyrolytic-carbon free–radical evolution and irradiation damage of polyimide under low energy proton-irradiation. J. Appl. Phys..

[13] Kang P.-H., Jeon Y.-K., Jeun J.-P., Shin J.-W., Nho Y.-C. (2008). Effect of electron beam irradiation on polyimide film. J. Ind. Eng. Chem..

[14] Alegaonkar P.S., Bhoraskar V.N. (2004). Effect of MeV electron irradiation on the free volume of polyimide. Radiat. Eff. Defects Solids.

[15] Dong S.-S., Shao W.-Z., Yang L., Ye H.-J., Zhen L. (2018). Microstructure evolution of polyimide films induced by electron beam irradiation-load coupling treatment. Polym. Degrad. Stab..

[16] Mishra R., Tripathy S.P., Dwivedi K.K., Khathing D.T., Ghosh S., Müller M., Fink D. (2003). Spectroscopic and thermal studies of electron irradiated polyimide. Radiat. Measur..

[17] Yue L., Wu Y., Sun C., Xiao J., Shi Y., Ma G., He S. (2012). Investigation on the radiation induced conductivity of space-applied polyimide under cyclic electron irradiation. Nucl. Instrum. Methods Phys. Res. Sect. B Beam Interact. Mater. At..

[18] Wu Y., Ju D., Wang H., Sun C., Wu Y., Cao Z., Tolochko O.V. (2022). Simulation of the Particle Transport Behaviors in Nanoporous Matter. Polymers.

[19] Wu Y., Ju D., Wang H., Zhao H., Sun C., Wu Y., Guo B., Wang Y. (2020). Modification of surface structure and mechanical properties in polyimide aerogel by low-energy proton implantation. Surf. Coat. Technol..

[20] Wu Y., Ju D., Liu Y., Zhao H., Wang H., Sun C., Wu Y., Cao Z., Guo B. (2020). Evaluation of radiation damage behavior in polyimide aerogel by infrared camera and photoacoustic spectroscopy. Polym. Test..

[21] Fang G., Li H., Liu J., Ni H., Yang H., Yang S. (2015). Intrinsically Atomic-oxygen-resistant POSS-containing Polyimide Aerogels: Synthesis and Characterization. Chem. Lett..

[22] Dissado L.A., Fothergill J.C. (1992). Stevens, Electrical Degradation and Breakdown in Polymers.

[23] Iizuka T.T.T. Generic PD Resistance Characteristics of Polymer Nanocomposites. Proceedings of the 2010 Annual Report Conference on Electrical Insulation and Dielectic Phenomena.

[24] (2022). Standard Test Method for Contamination Outgassing Characteristics of Spacecraft Materials.

[25] Diaham S.Z.S., Locatelli M.-L., Dinculescu S., Decup M., Lebey T. (2010). Dielectric Breakdown of Polyimide Films: Area, Thickness and Temperature Dependence. IEEE Trans. Dielectr. Electr. Insul..

